# Comparison of the polyphenolic profile and antibacterial activity of the leaves, fruits and flowers of *Rhododendron ambiguum* and *Rhododendron cinnabarinum*

**DOI:** 10.1186/s13104-017-2601-1

**Published:** 2017-07-20

**Authors:** Abhinandan Shrestha, Ahmed Rezk, Inamullah Hakeem Said, Victoria von Glasenapp, Rachelle Smith, Matthias S. Ullrich, Hartwig Schepker, Nikolai Kuhnert

**Affiliations:** 1 Department of Life Science & Chemistry, Jacobs University Bremen, Campus Ring 1, 28759 Bremen, Germany; 2 Stiftung Bremer Rhododendronpark, Deliusweg 40, 28359 Bremen, Germany

**Keywords:** *Rhododendron*, Polyphenols, Antibacterial, Leaves, Fruits, Flowers

## Abstract

**Background:**

*Rhododendron* species have been traditionally used in countries like China, Nepal, Russia and North America for treating human diseases. These species are known to be a good source of polyphenolic plant secondary plant metabolites. They are known to have beneficial health properties for humans and have been used to treat diseases like asthma, skin diseases. In this contribution we investigate the phenolic profile and antibacterial activity of extracts from several plant organs including for the first time from leaves of different development stages.

**Methods:**

In this study, the polyphenolic profile of fruits, flowers and leaves of different ages of *Rhododendron ambiguum* and *Rhododendron cinnabarinum* were studied by using HPLC–MS and compounds identified based on high resolution masses and identity of tandem mass spectra, UV/VIS spectra and retention times if compared to standards.

**Results:**

Fifty-nine different polyphenols including isomers were identified in these species by their fragmentation pattern and high resolution data. Also, the antibacterial activity of these parts (leaves, fruits and flowers) against gram-positive bacteria was studied.

**Conclusion:**

The leaves and fruits contained more polyphenols than the flowers. With the exception of flowers, the fruits and leaves of both species were also determined to have a significant antibacterial effect against four gram-positive bacteria.

**Electronic supplementary material:**

The online version of this article (doi:10.1186/s13104-017-2601-1) contains supplementary material, which is available to authorized users.

## Background

**Fig. 1:**
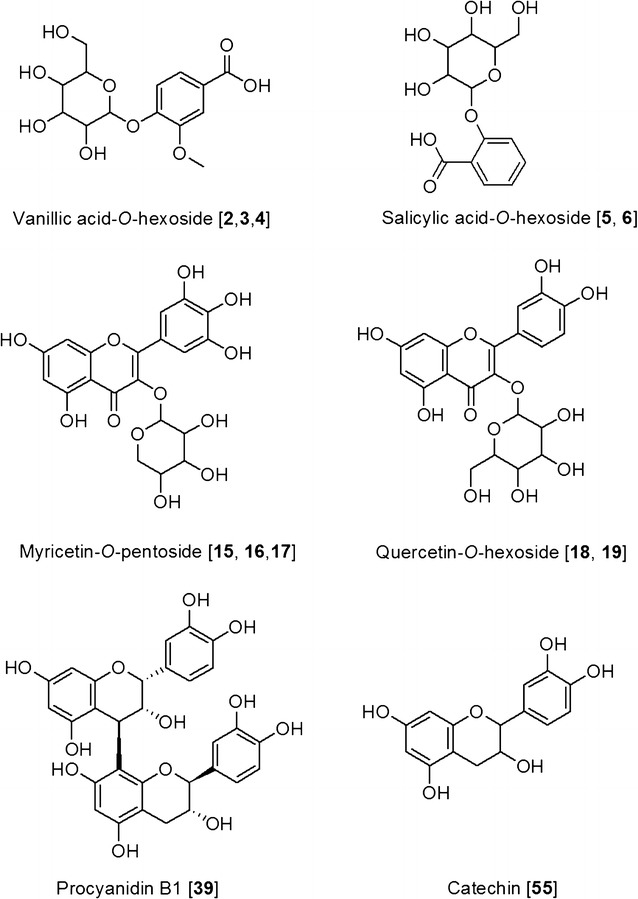
Representative examples of polyphenols from *Rhododendron* species. For the complete list of structures of compounds identified, please refer to Additional file 1

Polyphenols are secondary plant metabolites that are known to have different physical, chemical and biological properties. The four major types of polyphenols are flavonoids, phenolic acids, lignans and stilbenes. The phenolic acid polyphenols consist of the derivatives of hydroxybenzoic acid and hydroxycinnamic acid. The flavonoids are further classified into different groups based on their structure variation and hydroxylation pattern. Proanthocyanidins (PAs) belong to the class of flavonoids, which are formed from the oligomerization and polymerization of flavan-3-ol units such as catechin and afzelechin (Fig. 1). PAs are commonly found in fruits, vegetables and grain [1, 2]. They are known to have anti-inflammatory [3] and antimutagenic [4] effects and have been used in the treatment of asthma [4], skin diseases and UV radiations [5].

*Rhododendron* is a genus of woody plants that belong to Ericaceae family. Most of the species have attractive flowers and more than 1000 species of the genus have been described. They differ in their range of size, shape, texture, growth habit and color of blossoms [6]. The genus is ranging from shrubs and small to large trees. *Rhododendron* species have decorative flowers, which are mainly used for ornamental purposes. There is variation in the height of the plant, starting from 10 cm to 1 m (smallest species), while the example of largest specie is *R. giganteum*, which is almost 3 m tall. The leaves of most species are spirally arranged; the nature of leaves may be evergreen or deciduous.

The genus *Rhododendron* is found in almost all parts of the world except some parts in America and Africa. Species of the genus *Rhododendron* occur throughout the Northern Hemisphere and the Southern Hemisphere in South Eastern Asia and Northern Australasia. The plant is originally present in mountainous areas characterized by acidic well-drained soil, regular rainfall and cool summer temperatures [7]. On the other hand, majority of the genus prefers cooler temperature including the subgenus *Hymenanthes*. One of the subsections (*Pontica*) is indigenous to the areas outside the center of distribution and present in the areas of Japan, eastern China, Europe, North America and Russia [8].

*Rhododendron* (flowers and leaves) has traditionally been used in China, Nepal, North America, Russia, Korea, Austria and Romania for treating various diseases including arthritis, intestinal disorders, rheumatism, skin diseases, cough and other ailments [9]. They are known to be rich in polyphenolic compounds namely flavonoids and their glycosides, terpenoids and essential oils [1].

The new and exciting aspect of this study is the analysis of different plant organs where only limited data is available in the literature. The hypotheses of this study are that fruits, flowers and leaves have different polyphenolic profile and that the leaves of different ages, which are exposed to numerous environmental conditions might lead to the production of new compounds or variation in secondary metabolite quantities. In particular *Rhododendron* as an evergreen shrub grows a new generation of leaves every year offering the unique opportunity to compare the polyphenolic profile of leaves of different ages and hence study the biochemical history of the plant. Such an investigation has to the best of our knowledge never been carried out. The polyphenolic profile of the leaves of different age, flowers and fruits were analyzed for *R. ambiguum* and *R. cinnabarinum*. Both species belong to the subgenus *Rhododendron*, section *Rhododendron*. *R. ambiguum* and *R. cinnabarinum* belong to the subsection *Triflora* and *Cinnabarina* respectively. *R. cinnabarinum* is considered to be toxic for animals [9, 10]. It is known from our previous studies that these two species out of 17 *Rhododendron* species showed higher activity against several Gram-positive bacteria [11]. Moreover, the high dose of both *Rhododendron* species exhibited a toxic effect in two mammalian cells and induced phenotypic changes that are characteristic for apoptosis [12]. Thus, the aim of the study was to analyze the chemical profile of *Rhododendron* crude extracts in order to contribute to our on-going investigations on the bioactivity potential of *Rhododendron*.

## Methods

### Plant material and chemicals

Fresh leaf material of *R. ambiguum* Hemsley and *R. cinnabarinum* Hooker (first, second, and third leaf) were collected from plants grown in the Rhododendron-Park Bremen (http://www.rhododendronparkbremen.de) from April 2013 at 10:00 [gene bank number: *R. ambiguum* (100.007); *R. cinnabarinum* (100.322)]. First and second year leaves were collected for *R. ambiguum*, and first, second and third year leaves were collected for *R. cinnabarinum*. The leaves were distinguished on the basis of their morphological features. Moreover, the flowers and fruits for both *Rhododendron* species were also sampled. Each sample species was collected from three different individual plants with the help of Dr. Hartwig Schepker. The identities of all plant species have been authenticated according to the German Genebank Rhododendron Database provided by the Bundessortenamt (http://www.bundessortenamt.de/rhodo). Samples were deposited in herbaria with voucher numbers: *R. ambiguum*: OLD00801; *R. cinnabarinum*: OLD00757.

All chemicals (analytical grade) were purchased from Carl Roth (Karlsruhe, Germany). Standards were used whenever applicable, to compare the fragmentation and retention time of the compounds.

### Plant extraction

The *Rhododendron* leaves, fruits and flowers were freeze dried using liquid nitrogen. A mortar and pestle was used to crush the dried brittle leaves. 2 g of the powdered material were dissolved in 10 mL of 80% aqueous methanol and for 24 h at 4 °C. The mixture was then sonicated for 15 min and centrifuged at 3.220×*g* for 10 min. The aliquot was then separated and stored at −20 °C until further analyses.

### LC-ESI-TOF–MS (high resolution mass spectrometry)

The LC equipment (Agilent 1200 series, Bremen, Germany) consists of a binary pump, an auto-sampler with 100 µL loop and a UV–Vis detector with a light-pipe flow cell. The UV detector was used at 280 nm to measure the polyphenols. The 5 µm diphenyl column having 250 × 3 mm inner diameter (Varian, Darmstadt, Germany) was used for separation. This was connected to the microTOF mass spectrometer (Bruker Daltonics, Bremen, Germany) equipped with an electrospray ionization source. The internal calibration was achieved by using 0.1 M sodium formate solution at 0.10 mL/min, which was injected through the six-port valve. The calibration was achieved by using the enhanced quadratic mode. Water/formic acid (1000:0.05 v/v) and methanol were used as solvent A and B respectively. The flow rate of the solvents was adjusted to 500 µL/min. A linear gradient was used from 10% B to 80% B in 70 min and a further 10 min was assigned for the gradient to equilibrate from 80% B to 10% B for the next run. 3 µL of the filtered extract was injected into the system. The software used in this system was Bruker Hystar.

### LC-ESI-MS^*n*^ (Tandem mass spectrometry)

The Liquid chromatography equipment (Agilent 1100 series) comprises of a binary pump, an auto sampler having a 100 µL capacity loop and a Diode Array Detector with a range from 200 to 600 nm. The detector recorded at 254, 280 and 320 nm, which is the best absorption wavelength for polyphenolic compounds. Chromatographic separation was performed using the same gradient method used in the LC-TOF analyses. A 5 μm diphenyl column of 250 × 3 mm i.d. (Varian, Darmstadt, Germany) with 500 µL/min flow rate of solvent was used. The LC equipment was connected with Ion-trap mass spectrometer, which was fitted with an ESI source (Bruker Daltonics HCT Ultra, Bremen, Germany) operating in full scan auto MS^*n*^ mode to obtain fragment ions. Tandem mass spectra were acquired in Auto-MS^*n*^ mode (smart fragmentation) using a ramping of the collision energy. Maximum fragmentation amplitude was fixed to 1 V. MS operating conditions (negative mode) had been optimized with a capillary temperature of 365 °C, a dry gas flow rate was of 10 L/min, and a nebulizer pressure of 10 psi. 3 µL of the filtered extract was injected into the system. The software used in this system was Agilent Chemstation.

### Bacterial strains and antimicrobial susceptibility test

Four Gram-Positive bacterial species i.e. *Bacillus subtilis S168*, *Bacillus aquimaris* MB-2011, *Bacillus thioparus*, and *Clavibacter michiganensis* and one Gram-Negative bacterial specie i.e. *Escherichia coli* were selected to compare the susceptibility of crude extracts of leaves, flower and fruit of two *Rhododendron* species i.e. *R. ambiguum* and *R. cinnabarinum*. Antimicrobial activity screening was conducted by the agar diffusion method [13]. Briefly, Lysogeny Broth (LB) agar plates were inoculated with 200 µL of the inoculum of the tester organism (1 × 10^7^ colony forming units per mL) by evenly spreading the cell suspensions over the agar surface. Holes with diameters of 5 mm were punched into the agar plates. Subsequently, 50 µL of the plant crude extracts were filled into each well. The plates were incubated overnight at 28 °C. Inhibition of microbial growth was determined by measuring the radius of the inhibition zone. For each bacterial strain, 80% aqueous methanol solutions were used as negative solvent controls. All experiments were performed in triplicates and the results were presented as mean values. Ampicillin was used as the positive control and the extraction solvent, 80% aqueous methanol was used as the negative control.

## Results and discussion

To our knowledge, no investigation on the polyphenolic profile of leaves of different ages has been carried out. The crude extracts of all parts of the two selected species *R. ambiguum* and *R*. *cinnabarinum* (i.e. first, second, third leaves, flowers and fruits) were extracted with 80% aqueous methanol in order to extract polyphenols. Both species were recently shown to possess promising biological activities [11] and their plants consisted of leaves of different age. These extracts were analyzed by reversed phase HPLC using a diphenyl column with a gradient using methanol and water/formic acid (1000:0.05 v/v). Negative ion mode was used to study the polyphenols using tandem mass spectrometry and high resolution mass spectrometry. The compounds were identified in the high resolution mass data by observing an absolute mass error below 5 ppm for their elemental composition. The UV spectrum at 280 nm was used to identify the PAs present in the samples. Also, the fragmentation pathway of PAs by heterocyclic ring fission (HRF) and retro-Diels–Alder (RDA) reaction were considered [14].

### Total ion chromatograms from LC–MS

**Fig. 2:**
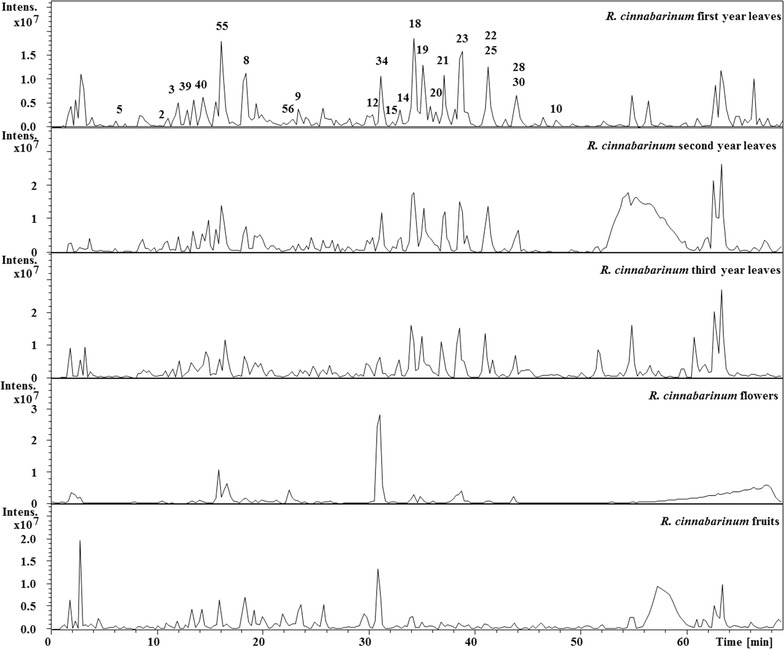
Total Ion Chromatogram of *Rhododendron cinnabarinum* leaves, flowers and fruits by LC-MS^*n*^ in negative mode

The chromatograms of *Rhododendron cinnabarinum* first year leaves, second year leaves, third year leaves, flowers and fruits were directly compared and are shown in Fig. 2. The chromatograms of the first, second and third year leaves consisted of peaks at identical retention time and comparable intensities. The chromatograms of flowers and fruits were different compared to the leaves. The chromatogram of flowers consisted of peaks with lower intensity in the region 0–30 min, which suggests that the hydrophilic compounds are present in lower intensity compared to the leaves. However, after 30 min, the chromatogram of the flowers was similar to the leaves. On the other hand, the chromatogram of the fruits exhibited additional peaks not present in the leaves and flowers.

In this study, 59 polyphenolic compounds were identified from both *Rhododendron* species for the first, second third year leaves, flower and fruit (Table 1). The identification of reported compounds was based on their retention time, fragmentation pattern and high resolution mass data. All identified polyphenol compounds have been discovered in nature already [1, 15–26]. Out of the identified compounds, 46 were already reported to be found in *Rhododendron* species [1, 16–18]. In this study, 13 new polyphenolic compounds were identified in *Rhododendron species*. The fruits consisted of the highest variety and concentration of PAs among the four parts of *R. ambiguum* and *R. cinnabarinum*.

**Table 1:** Polyphenols present in different year leaves, flowers and fruits of *R. ambiguum* and *R. cinnabarinum*

No.	Species	References	*m/z* [M–H]^−^	RT	*R. ambiguum*				*R. cinnabarinum*				
	Part				1st	2nd	Fl.	Fr.	1st	2nd	3rd	Fl.	Fr.
	Compound												
1	Methyl gallate hexoside	[35]	345.0827	11.8		Y							
2	Vanillic acid-*O*-hexoside	[19]	329.0864	10.2		Y	Y	Y	Y	Y	Y	Y	Y
3	Vanillic acid-*O*-hexoside	[19]	329.0867	11.7	Y	Y	Y	Y	Y	Y			Y
4	Vanillic acid-*O*-hexoside	[19]	329.0864	13.9					Y	Y	Y	Y	
5	Salicylic acid-*O*-hexoside	[20]	299.0761	6.3		Y			Y	Y	Y	Y	Y
6	Salicylic acid-*O*-hexoside	[20]	299.0762	8.5						Y			
7	3-*O*-Caffeoylquinic acid	[1]	353.0877	13.0	Y	Y	Y	Y			Y		
8	5-*O*-Caffeoylquinic acid	[1]	353.0873	18.2		Y	Y	Y	Y	Y	Y		Y
9	4-*O*-Caffeoylquinic acid	[1]	353.864	23.0		Y			Y	Y	Y		
10	Naringenin	[29]	271.0602	47.9	Y	Y		Y	Y			Y	Y
11	Myricetin	[29]	317.0296	38.9	Y	Y	Y	Y					Y
12	Myricetin-*O*-hexoside	[1]	479.0820	30.8			Y	Y	Y	Y	Y		Y
13	Myricetin-*O*-hexoside	[1]	479.0853	35.3	Y	Y	Y	Y					
14	Myricetin-*O*-rhamnoside	[1]	463.0899	33.0					Y	Y	Y		Y
15	Myricetin-*O*-pentoside	[1]	449.0728	32.1				Y	Y	Y	Y		Y
16	Myricetin-*O*-pentoside	[1]	449.0725	37.8				Y	Y	Y			
17	Myricetin-*O*-pentoside	[1]	449.0732	39.1	Y	Y	Y	Y					
18	Quercetin-*O*-hexoside	[1]	463.0898	34.2	Y	Y	Y	Y	Y	Y	Y	Y	Y
19	Quercetin-*O*-hexoside	[1]	463.0897	35.5	Y	Y		Y		Y	Y	Y	Y
20	Quercetin-*O*-pentoside	[1]	433.0771	36.6	Y	Y			Y	Y	Y		
21	Quercetin-*O*-pentoside	[1]	433.0795	37.4	Y	Y	Y	Y	Y	Y	Y	Y	Y
22	Quercetin-*O*-pentoside	[1]	433.0798	41.2	Y	Y	Y	Y	Y	Y	Y	Y	Y
23	Quercetin-*O*-rhamnoside	[1]	447.0921	38.7	Y	Y	Y	Y	Y	Y	Y	Y	Y
24	Quercetin-*O*-rhamnoside-*O*-hexoside	[1]	609.1442	34.3	Y	Y			Y	Y			
25	Quercetin-*O*-glucoronide	[21]	477.0676	40.8						Y	Y		Y
26	Quercetin	[1]	301.0342	44.4	Y	Y	Y	Y		Y			
27	Kamepferol	[29]	285.0414	49.5	Y	Y	Y	Y					
28	Kaempferol-3-*O*-rhamnoside	[22]	431.0982	43.6	Y	Y	Y		Y	Y	Y	Y	Y
29	Kaempferol-3-*O*-pentoside	[22]	417.0827	41.0		Y	Y	Y					
30	Kaempferol-3-*O*-pentoside	[22]	417.0833	43.9		Y	Y	Y	Y	Y	Y	Y	Y
31	Kaempferol-3-*O*-glucoronide	[23]	461.0719	43.6						Y			
32	Taxifolin	[36]	303.0507	29.1		Y	Y						
33	Taxifolin-*O*-pentoside	[1]	435.0936	27.8	Y	Y		Y					Y
34	Taxifolin-*O*-pentoside	[1]	435.0936	31.1	Y	Y	Y	Y	Y	Y	Y	Y	Y
35	Taxifolin-*O*-pentoside	[1]	435.0930	33.6		Y							
36	(Epi)gallocatechin-(epi)gallocatechin	[1]	609.1259	6.5				Y					Y
37	(Epi)gallocatechin-(epi)gallocatechin	[1]	609.1246	7.5				Y					
38	(Epi)gallocatechin-(epi)gallocatechin	[1]	609.1251	10.2				Y					
39	(Epi)catechin-(epi)catechin (Procyanidin dimer B1)	[1]	577.1372	13.4	Y	Y	Y	Y	Y	Y	Y	Y	Y
40	(Epi)catechin-(epi)catechin (Procyanidin dimer B)	[1]	577.1377	14.3	Y	Y	Y	Y	Y		Y	Y	Y
41	(Epi)catechin-(epi)catechin (Procyanidin dimer B)	[1]	577.1373	18.6			Y	Y				Y	Y
42	(Epi)catechin-(epi)catechin (Procyanidin dimer B2)	[1]	577.1367	20.3		Y	Y	Y	Y	Y	Y	Y	Y
43	(Epi)catechin-(epi)catechin (Procyanidin dimer B)	[1]	577.1358	23.1			Y	Y	Y	Y	Y		Y
44	Procyanidin Trimer C	[24]	865.1994	5.6		Y		Y	Y	Y	Y		Y
45	Procyanidin Trimer C	[24]	865.1953	25.6				Y					
46	A type Procyanidin Trimer C	[24]	863.1805	22.2					Y	Y	Y	Y	Y
47	(Epi)gallocatechin-(epi)catechin	[1]	593.1310	7.7				Y	Y	Y			Y
48	(Epi)gallocatechin-(epi)catechin	[1]	593.1309	9.7			Y	Y	Y	Y	Y		Y
49	(Epi)gallocatechin-(epi)catechin	[1]	593.1314	10.5				Y					Y
50	(Epi)gallocatechin-(epi)catechin	[1]	593.1307	12.6				Y					Y
51	(Epi)gallocatechin-(epi)catechin	[1]	593.1323	13.7			Y	Y					Y
52	(Epi)gallocatechin-(epi)catechin	[1]	593.1311	18.2				Y					
53	(Epi)catechin-(4,8/2,6)-(epi)catechin	[1]	575.1209	27.2	Y	Y	Y	Y					Y
54	(Epi)catechin-(4,8/2,6)-(epi)catechin	[1]	575.1201	32.4	Y	Y	Y	Y					Y
55	Catechin	[1]	289.0721	16.0	Y	Y	Y	Y	Y	Y	Y	Y	Y
56	Epicatechin	[1]	289.0713	23.0	Y	Y	Y	Y	Y			Y	
57	Gallocatechin	[1]	305.0656	8.5		Y	Y	Y					Y
58	Epigallocatechin	[1]	305.0660	15.4		Y	Y	Y					
59	(Epi)catechin-*O*-D-glucopyranoside	[25]	451.1258	10.5					Y	Y	Y		Y

*RT* retention time, *Fl.* flowers, *Fr.* Fruits, *1st, 2nd, 3rd* 1st, 2nd, 3rd year leaves

#### Characterization of methyl gallate hexoside [1] (M_r_ 345)

A peak was detected at *m/z* 345 and was assigned to be methyl gallate hexoside. The peak produced the fragmentation with base peak at *m/z* 183 and secondary peaks of *m/z* 168 and 124.

#### Characterization of (epi)catechin-(4,8′/2,6′)-(epi)catechin [53, 54] (M_r_ 576)

Two peaks were detected at *m/z* 575 and were assigned as A-type dimer of (epi)catechin unit. The first peak produced an MS^2^ base peak of *m/z* 449 and secondary peak of *m/z* 287. The second peak produced an MS^2^ base peak of *m/z* 423 and secondary peak of *m/z* 285.

#### Characterization of catechin [55] and epicatechin [56] (M_r_ 290)

Two peaks were detected at *m/z* 289. The first peak was assigned as catechin and the second peak was assigned as epicatechin based on their polarity. Both peaks produced similar fragmentation, consisting of an MS^2^ base peak of *m/z* 245 and secondary peak of *m/z* 203.

#### Characterization of gallocatechin [57] and epigallocatechin [58] (M_r_ 306)

Two peaks were detected at *m/z* 305. The first peak was assigned as gallocatechin and the second peak was assigned as epigallocatechin based on their polarity. Both peaks produced similar fragmentation, consisting of an MS^2^ base peak of *m/z* 179 and secondary peak of *m/z* 164.

#### Characterization of (epi)catechin-(4,8′)-(epi)catechin [39, 40, 41, 42, 43] (M_r_ 578)

Five peaks were detected at *m/z* 577. They were assigned as the PA dimer. All the peaks produced similar fragmentation with the base peak of *m/z* 407 and secondary peaks of *m/z* 425 and 285. The standards of the dimers B1 and B2 were used to differentiate the isomers by their retention time.

#### Characterization of (epi)gallocatechin-(4,8′)-(epi)catechin and (epi)catechin-(4,8′)-(epi)gallocatechin [47, 48, 49, 50, 51, 52] (M_r_ 594)

Six peaks were detected at *m/z* 593. They were speculated to be dimeric B-type PA consisting of (epi)catechin and (epi)gallocatechin monomeric units. All the peaks produced similar fragmentation having the base peak of *m/z* 425 and secondary peak of *m/z* 407.

#### Characterization of (epi)gallocatechin-(4,8′)-(epi)gallocatechin [36, 37, 38] (M_r_ 610)

Three peaks were detected at *m/z* 609 and were speculated to be dimeric B-type PAs with (epi)gallocatechin monomeric units. The three peaks produced the fragmentation with base peak at *m/z* 423 and secondary peaks of *m/z* 441 and 283.

#### Characterization of Taxifolin [27] (M_r_ 304)

A peak was detected at *m/z* 303 and was assigned to be taxifolin. The peak produced the fragmentation with base peak at *m/z* 285 and secondary peaks of *m/z* 177 and 125.

### Identification of other polyphenols

**Fig. 3:**
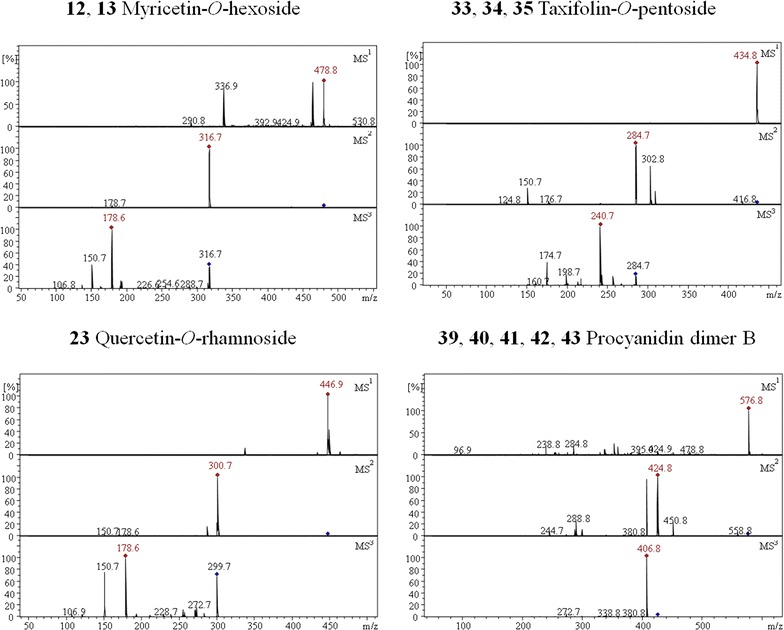
Tandem MS fragmentation of some polyphenols identified in *Rhododendron*

The polyphenols were identified by their specific fragmentation patterns, retention time and high resolution mass values. The other polyphenols that were identified in the leaf extracts are three vanillic acid-*O*-hexosides [19], two salicylic acid hexosides [20, 28], three caffeoylquinic acids [1], naringenin [29], myricetin [29], two myricetin-*O*-hexosides [1], myricetin-*O*-rhamnoside [1], three myricetin-*O*-pentosides [1], two quercetin-*O*-hexosides [1], three quercetin-*O*-pentosides [1], quercetin-*O*-rhamnoside [1], quercetin-*O*-rhamnoside-*O*-hexoside [1], quercetin-*O*-glucoronide [21, 30], quercetin [1], kaempferol [27, 29], kaempferol-*O*-rhamnoside [22], two kaempferol-*O*-pentosides [22], kaempferol-*O*-glucoronide [23, 30], three taxifolin-*O*-pentosides [1, 31], two procyanidin trimers C [25, 32], one A-type procyandintrimer C [21, 24] and (epi)catechin-*O*-d-glycopyranoside [25]. The tandem mass spectra of some compounds are shown in Fig. 3.

### Antibacterial activity

**Fig. 4:**
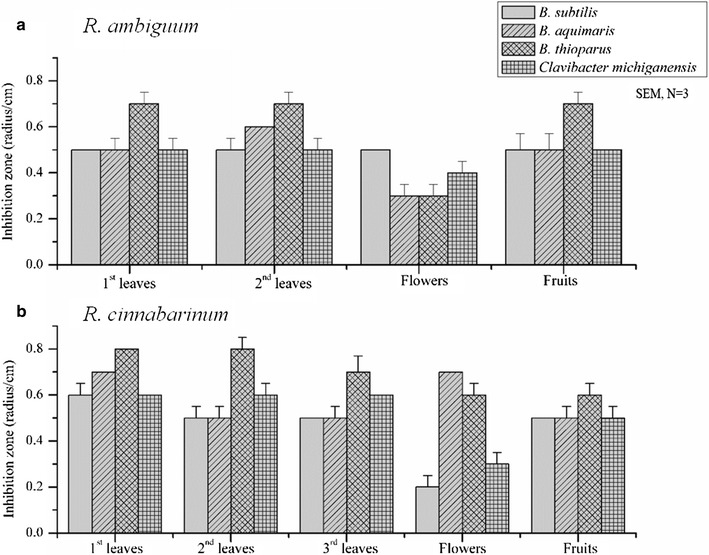
Antimicrobial activities of methanol crude extract of different plant parts. **a** *R. ambiguum* and **b** *R. cinnabarinum*

Four Gram-positive organisms were used to compare the antibacterial activity of different plant parts of two species from genus *Rhododendron*. Crude extract of first, second, third year leaves in addition to flowers and fruits of *R. ambiguum* and *R. cinnabarinum* were obtained using 80% methanol. There was no bioactivity observed for crude extracts of all samples against *Escherichia coli*. Against Gram-positive bacteria, the bioactivity of *R. ambiguum* ranged between 0.5 and 0.7 cm, while for *R. cinnabarinum* between 0.5 and 0.8 cm (Fig. 4). Antibacterial effects of fruit and leaves extracts were in the same order of magnitude. However, there was a reduced antibacterial activity observed for the flowers of *R. cinnabarinum* and *R. ambiguum*. This could be due to the evolutionary aspect as flowers have a short blooming period in a year compared to the leaves and fruits. In general, *B. thioparus* was the most sensitive bacteria species towards the plant parts for both *Rhododendron* species. These results are in agreement with our previous study, which showed a higher antibacterial effect of *Rhododendron* species against Gram-positive and higher effect for *R. cinnabarinum* [11]. The polyphenolic analysis indicated that Taxifolin derivatives were present in high concentration in the leaves of both plant species, which could be the reason of apoptosis like phenotype observed before in other studies [12]. Moreover, this finding is also supported by other studies, which reported the effect of Taxifolin in different cancer cell lines by inducing apoptosis cell death [33, 34].

The radius of the inhibition zones was measured in triplicates and the values are given as means ± standard deviations. The aqueous methanol used as negative controls did not yield inhibition zones (data not shown).

Since flowers were found to have lower bioactivity as compared to the leaves and fruits, a list of compounds present in either leaves or fruits, but not in flowers was compiled. This list consisted of **1** (methyl gallate hexoside), **6 **(salicylic acid-*O*-hexoside), **9** (4-*O*-caffeoylquinic acid), **14 **(myricetin-*O*-rhamnoside), **15**, **16** (myricetin-*O*-pentoside), **20** (quercetin-*O*-pentoside), **24 **(quercetin-*O*-rhamnoside-*O*-hexoside), **31** (kaempferol-3-*O*-glucuronide), **33**, **35** (taxifolin-*O*-pentoside), **36**, **37**, **38** (epi)gallocatechin-(epi)gallocatechin, **44**, **45** (procyanidin trimer C), **47**, **49**, **50**, **52** ((epi)gallocatechin-(epi)catechin) and **59** ((epi)catechin-*O*-D-glucopyranoside). The presence of these compounds in the plant organs could be either a contributing factor to the bioactivity itself or could function as a marker for plant organs with potential bioactivity.

## Conclusions

The different parts of the *R. ambiguum* and *R. cinnabarinum* are a rich source of polyphenols. Fifty-nine different types of polyphenols including isomers were identified in these parts based on their fragmentation pattern and high resolution mass spectra in negative ion mode. The polyphenolic profile of different year leaves was found to be similar. Among all the parts, the fruits were found to contain the highest variety and concentration of polyphenols. PAs were mainly found in the fruits. However, there are many unidentified compounds present in leaves, flowers and fruits, which need to be analyzed in future. We can conclude that both *Rhododendron* species have antibacterial effect towards Gram-positive bacteria, while there was no significant difference between different seasonal leaves and fruits, but low effect for flowers.

## Additional file

**Additional file 1: Figure A1.** Total Ion Chromatogram of *Rhododendron ambiguum* first year leaves, second year leaves, flowers and fruits generated by LC-MS^*n*^ in negative mode. **Figure A2.** Structures of all identified compounds in *Rhododendron ambiguum* and *Rhododendron cinnabarinum.* **Table A1.** Fragmentation pattern of the identified compounds in *Rhododendron ambiguum* and *Rhododendron cinnabarinum.*

## Abbreviations

ESI: electro spray ionization
HPLC: high performance liquid chromatography
LB: Lysogeny Broth
LC: liquid chromatography
*m/z*: mass to charge ratio
M: molar
M_r_: molecular weight
MS: mass spectrometry
MS^*n*^: tandem mass spectrometry
PAs: proanthocyanidins
psi: pound-force per square inch
RT: retention time
TOF: time of flight
UV: ultra violet
v/v: volume/volume
Vis: visible
