# Supplementary material for: Comparison of the polyphenolic profile and antibacterial activity of the leaves, fruits and flowers of Rhododendron ambiguum and Rhododendron cinnabarinum
Source: BMC Res Notes. 2017 Jul 20;10:297. doi: 10.1186/s13104-017-2601-1 (PMC5520227; doi:10.1186/s13104-017-2601-1)
Supplement: Supplementary file 1 — Additional file 1: Figure A1. Total Ion Chromatogram of Rhododendron ambiguum first year leaves, second year leaves, flowers and fruits generated by LC-MSn in negative mode. Figure A2. Structures of all identified compounds in Rhododendron ambiguum and Rhododendron cinnabarinum. Table A1. Fragmentation pattern of the identified compounds in Rhododendron ambiguum and Rhododendron cinnabarinum. [file 13104_2017_2601_MOESM1_ESM.docx]

*R. ambiguum* first year leaves

*R. ambiguum* second year leaves

*R. ambiguum* flowers

*R. ambiguum* fruits

0

1

2

3

7

x10

Intens.

0

1

2

3

4

7

x10

Intens.

0

1

2

3

7

x10

Intens.

0

1

2

3

7

x10

Intens.

0

10

20

30

40

50

60

Time [min]

**Figure A1 Total Ion Chromatogram of *Rhododendron ambiguum* first year leaves, second year leaves, flowers and fruits generated by LC-MS*^n^* in negative mode**

**Figure A2 Structures of all identified compounds in *Rhododendron ambiguum* and *Rhododendron cinnabarinum***

**1 Methyl gallatehexoside**

**2, 3, 4 Vanillic acid-*O*-hexoside**

**5, 6 Salicylic acid*-O-*hexoside**

**7 3*-O-*Caffeoylquinic acid**

**8 5*-O-*Caffeoylquinic acid**

**9 4*-O-*Caffeoylquinic acid**

**10 Naringenin**

**11 Myricetin**

**12, 13 Myricetin*-O-*hexoside**

**14 Myricetin*-O-*rhamnoside**

**15, 16, 17 Myricetin*-O-*pentoside**

**18, 19 Quercetin*-O-*hexoside**

**20, 21, 22 Quercetin*-O-*pentoside**

**23 Quercetin*-O-*rhamnoside**

**24 Quercetin*-O-*rhamnoside*-O-*hexoside**

**25 Quercetin-*O*-glucoronide**

**26 Quercetin**

**27 Kamepferol**

**28 Kaempferol-3*-O-*rhamnoside**

**29, 30 Kaempferol-3*-O-*pentoside**

**31 Kaempferol-3*-O-*glucoronide**

**32 Taxifolin**

**33, 34, 35 Taxifolin*-O-*pentoside**

**36, 37, 38 (Epi)gallocatechin-(epi)gallocatechin**

**39 (Epi)catechin-(epi)catechin (Procyanidin dimer B1)**

**40, 41, 43 (Epi)catechin-(epi)catechin (Procyanidin dimer B)**

**42 (Epi)catechin-(epi)catechin (Procyanidin dimer B2)**

**44, 45, 46 ProcyanidinTrimer C**

**47, 48, 49, 50, 51, 52 (Epi)gallocatechin-(epi)catechin**

**53, 54 (Epi)catechin-(4,8/2,6)-(epi)catechin**

**55 Catechin**

**56 Epicatechin**

**57 Gallocatechin**

**58 Epigallocatechin**

**59 (Epi)catechin*-O-*D-glucopyranoside**

**Table A1 Fragmentation pattern of the identified compounds in *Rhododendron ambiguum* and *Rhododendron cinnabarinum***

| **No.** | **Compound** | **Parent Ion [M-H]^-^**  ***m/z*** | **Retention Time**  **(min)** | **Fragmentation** |
| --- | --- | --- | --- | --- |
| 1 | Methyl gallatehexoside | 345.0827 | 11.8 | MS^2^🡪 183 (100), 168 (5), 124 (5) |
| 2 | Vanillic acid hexoside | 329.0864 | 10.2 | MS^2^🡪 167 (100); MS^3^🡪 152 (100), 123 (81) |
| 3 | Vanillic acid hexoside | 329.0867 | 11.7 | MS^2^🡪 167 (100); MS^3^🡪 149 (100), 123 (3) |
| 4 | Vanillic acid hexoside | 329.0864 | 13.9 | MS^2^🡪 167 (100); MS^3^🡪 123 (100), 152 (17) |
| 5 | Salicylic acid-*O*-hexoside | 299.0761 | 6.3 | MS^2^🡪 137 (100); MS^3^🡪 93 (100) |
| 6 | Salicylic acid-*O*-hexoside | 299.0762 | 8.5 | MS^2^🡪 137 (100); MS^3^🡪 93 (100) |
| 7 | 3-*O*-Caffeoylquinic acid | 353.0877 | 13.0 | MS^2^🡪 191 (100), 179 (38) |
| 8 | 5-*O*-Caffeoylquinic acid | 353.0873 | 18.2 | MS^2^🡪191 (100) |
| 9 | 4-*O*-Caffeoylquinic acid | 353.864 | 23.0 | MS^2^🡪173 (100) |
| 10 | Naringenin | 271.0602 | 47.9 | MS^2^🡪151 (100), 177 (25) |
| 11 | Myricetin | 317.0296 | 38.9 | MS^2^🡪179 (100), 151 (37) |
| 12 | Myricetin-*O*-hexoside | 479.0820 | 30.8 | MS^2^🡪317 (100); MS^3^🡪 179 (100), 151 (31) |
| 13 | Myricetin-*O*-hexoside | 479.0853 | 35.3 | MS^2^🡪316 (100), 317 (80); MS^3^🡪 271 (100), 179 (61), 151 (21) |
| 14 | Myricetin-*O*-rhamnoside | 463.0899 | 33.0 | MS^2^🡪316 (100), 317 (73); MS^3^🡪 271 (100), 179 (60), 151 (22) |
| 15 | Myricetin-*O*-pentoside | 449.0728 | 32.1 | MS^2^🡪317 (100); MS^3^🡪 179 (100), 151 (30) |
| 16 | Myricetin-*O*-pentoside | 449.0725 | 37.8 | MS^2^🡪316 (100); MS^3^🡪 271 (100), 179 (26), 151 (11) |
| 17 | Myricetin-*O*-pentoside | 449.0732 | 39.1 | MS^2^🡪317 (100); MS^3^🡪 179 (100), 151 (35) |
| 18 | Quercetin-*O*-hexoside | 463.0898 | 34.2 | MS^2^🡪301 (100); MS^3^🡪 179 (100), 151 (80) |
| 19 | Quercetin-*O*-hexoside | 463.0897 | 35.5 | MS^2^🡪301 (100); MS^3^🡪 179 (100), 151 (73) |
| 20 | Quercetin-*O*-pentoside | 433.0771 | 36.6 | MS^2^🡪301 (100); MS^3^🡪 179 (100), 151 (13) |
| 21 | Quercetin-*O*-pentoside | 433.0795 | 37.4 | MS^2^🡪301 (100); MS^3^🡪 179 (100), 151 (68) |
| 22 | Quercetin-*O*-pentoside | 433.0798 | 41.2 | MS^2^🡪301 (100); MS^3^🡪 271 (100), 179 (100), 151 (37) |
| 23 | Quercetin-*O*-rhamnoside | 447.0921 | 38.7 | MS^2^🡪301 (100); MS^3^🡪 179 (100), 151 (67) |
| 24 | Quercetin-*O*-rhamnoside-*O*-hexoside | 609.1442 | 34.3 | MS^2^🡪301 (100); MS^3^🡪 179 (100), 151 (71) |
| 25 | Quercetin-*O*-glucoronide | 477.0676 | 40.8 | MS^2^🡪301 (100); MS^3^🡪 179 (100), 151 (85) |
| 26 | Quercetin | 301.0342 | 44.4 | MS^2^🡪179 (100), 151 (87) |
| 27 | Kamepferol | 285.0414 | 49.5 | MS^2^🡪151 (100) |
| 28 | Kaempferol-*O*-rhamnoside | 431.0982 | 43.6 | MS^2^🡪285 (100), 255 (9) |
| 29 | Kaempferol-*O*-pentoside | 417.0827 | 41.0 | MS^2^🡪285 (100), 255 (8) |
| 30 | Kaempferol-*O*-pentoside | 417.0833 | 43.9 | MS^2^🡪285 (100), 255 (16) |
| 31 | Kaempferol-*O*-glucoronide | 461.0719 | 43.6 | MS^2^🡪285 (100), 257 (2) |
| 32 | Taxifolin | 303.0507 | 29.1 | MS^2^🡪285 (100), 177 (13), 125 (12) |
| 33 | Taxifolin-*O*-pentoside | 435.0936 | 27.8 | MS^2^🡪285 (100), 303 (65); MS^3^🡪 241 (100), 175 (53) |
| 34 | Taxifolin-*O*-pentoside | 435.0936 | 31.1 | MS^2^🡪285 (100), 303 (65); MS^3^🡪 241 (100), 175 (38) |
| 35 | Taxifolin-*O*-pentoside | 435.0930 | 33.6 | MS^2^🡪285 (100), 303 (63); MS^3^🡪 241 (100), 175 (63) |
| 36 | (Epi)gallocatechin-(epi)gallocatechin | 609.1259 | 6.5 | MS^2^🡪423 (100), 441 (81), 305 (35); MS^3^🡪 283 (100), 297 (89), 255 (31) |
| 37 | (Epi)gallocatechin-(epi)gallocatechin | 609.1246 | 7.5 | MS^2^🡪423 (100), 441 (69), 305 (26); MS^3^🡪 283 (100), 297 (80), 255 (35) |
| 38 | (Epi)gallocatechin-(epi)gallocatechin | 609.1251 | 10.2 | MS^2^🡪423 (100), 441 (75), 305 (37); MS^3^🡪 283 (100), 297 (97), 255 (28) |
| 39 | (Epi)catechin-(epi)catechin (Procyanidin dimer B1) | 577.1372 | 13.4 | MS^2^🡪407 (100), 425 (97), 451 (34), 289 (34); MS^3^🡪 285 (100), 283 (35) |
| 40 | (Epi)catechin-(epi)catechin (Procyanidin dimer B) | 577.1377 | 14.3 | MS^2^🡪425 (100), 407 (93), 451 (20), 289 (22); MS^3^🡪 407 (100); MS^4^🡪 285 (100), 283 (27) |
| 41 | (Epi)catechin-(epi)catechin (Procyanidin dimer B) | 577.1373 | 18.6 | MS^2^🡪425 (100), 407 (93), 451 (32), 289 (40); MS^3^🡪 285 (100), 283 (36) |
| 42 | (Epi)catechin-(epi)catechin (Procyanidin dimer B2) | 577.1367 | 20.3 | MS^2^🡪425 (100), 407 (80), 451 (23), 289 (21); MS^3^🡪 407 (100); MS^4^🡪 285 (100), 283 (29) |
| 43 | (Epi)catechin-(epi)catechin (Procyanidin dimer B) | 577.1358 | 23.1 | MS^2^🡪425 (100), 407 (85), 451 (17), 289 (23); MS^3^🡪 407 (100); MS^4^🡪 285 (100), 283 (25) |
| 44 | Procyanidin Trimer C | 865.1994 | 5.6 | MS^2^🡪695 (100), 577 (37), 575 (33), 543 (25), 451 (15), 425 (14), 407 (25), 287 (27); MS^3^🡪 243 (100), 543 (73), 525 (41), 451 (47), 405 (46), 289 (24) |
| 45 | Procyanidin Trimer C | 865.1953 | 25.6 | MS^2^🡪695 (100), 577 (65), 575 (31), 543 (26), 451 (20), 425 (24), 407 (62), 287 (26); MS^3^🡪 543 (100), 525 (38), 451 (19), 405 (21), 289 (15), 283 (13), 243 (41) |
| 46 | A type Procyanidin Trimer C | 863.1805 | 22.2 | MS^2^🡪 711 (100), 693 (45), 559 (31), 541 (13), 451 (25), 411 (44), 289 (13); MS^3^🡪 693 (100), 559 (72), 541 (29), 407 (20) |
| 47 | (Epi)gallocatechin-(epi)catechin | 593.1310 | 7.7 | MS^2^🡪425 (100), 407 (76), 289 (30); MS^3^🡪 407 (100); MS^4^🡪 285 (100), 283 (18) |
| 48 | (Epi)gallocatechin-(epi)catechin | 593.1309 | 9.7 | MS^2^🡪425 (100), 407 (61), 289 (18); MS^3^🡪 407 (100); MS^4^🡪 285 (100), 283 (35) |
| 49 | (Epi)gallocatechin-(epi)catechin | 593.1314 | 10.5 | MS^2^🡪423 (100), 441 (48), 467 (32), 305 (41); MS^3^🡪 283 (100), 297 (78) |
| 50 | (Epi)gallocatechin-(epi)catechin | 593.1307 | 12.6 | MS^2^🡪425 (100), 407 (74), 289 (20); MS^3^🡪 407 (100); MS^4^🡪 285 (100), 283 (38) |
| 51 | (Epi)gallocatechin-(epi)catechin | 593.1323 | 13.7 | MS^2^🡪425 (100), 407 (70), 289 (22); MS^3^🡪 407 (100); MS^4^🡪 285 (100), 283 (30) |
| 52 | (Epi)gallocatechin-(epi)catechin | 593.1311 | 18.2 | MS^2^🡪425 (100), 407 (85), 289 (20); MS^3^🡪 407 (100); MS^4^🡪 285 (100), 283 (41) |
| 53 | (Epi)catechin-(4,8/2,6)-(epi)catechin | 575.1209 | 27.2 | MS^2^🡪449 (100), 539 (36), 423 (19), 407 (32), 289 (53), 285 (39); MS^3^🡪 287 (100), 313 (26), 245 (13) |
| 54 | (Epi)catechin-(4,8/2,6)-(epi)catechin | 575.1201 | 32.4 | MS^2^🡪423 (100), 449 (100), 539 (17), 407 (13) 289 (27), 285 (31); MS^3^🡪 285 (100), 313 (18) |
| 55 | Catechin | 289.0721 | 16.0 | MS^2^🡪245 (100), 205 (35), 179 (12); MS^3^🡪 203 (100), 227 (23), 187 (14), 161 (18) |
| 56 | Epicatechin | 289.0713 | 23.0 | MS^2^🡪245 (100), 205 (41), 179 (17); MS^3^🡪 203 (100), 227 (22), 187 (21), 161 (19) |
| 57 | Gallocatechin | 305.0656 | 8.5 | MS^2^🡪179 (100), 221 (76), 261 (30); MS^3^🡪 164 (100), 151 (23), 135 (31) |
| 58 | Epigallocatechin | 305.0660 | 15.4 | MS^2^🡪179 (100), 221 (82), 261 (24); MS^3^🡪 164 (100), 151 (39), 135 (33) |
| 59 | (Epi)catechin-*O*-D-glucopyranoside | 451.1258 | 10.5 | MS^2^🡪 289 (100); MS^3^🡪 245 (100), 205 (47) |
